# Development and external validation of a clinical-genetic risk identification model for rheumatoid arthritis-associated interstitial lung disease

**DOI:** 10.3389/fimmu.2026.1885469

**Published:** 2026-09-03

**Authors:** Wei Fan, Wei Bo, Xuanhua Yu, Xiaojuan Gao, Jinmei Huang, Yi Zhang, Shufan Wu, Xuyan Chen, Liangjing Lu, Hanlin Yin

**Affiliations:** 1 Department of Rheumatology, Renji Hosptial, Shanghai Jiao Tong University School of Medicine, Shanghai, China; 2 Department of Rheumatology and Immunology, The Second Affiliated Hospital of Xiamen Medical College, Xiamen, China; 3 Department of Rheumatology and Immunology, Zhongshan Hospital of Xiamen University, School of Medicine, Xiamen University, Xiamen, China; 4 Department of Rheumatology, The Affiliated People’s Hospital of Fujian University of Traditional Chinese Medicine, Fuzhou, China; 5 Department of Rheumatology, Ningde Clinical Medical College of Fujian Medical University, Ningde, China

**Keywords:** interstitial lung disease, MUC5B, rheumatoid arthritis, risk factors, risk identification model

## Abstract

**Objective:**

This study aimed to develop and externally validate a clinical risk identification model for prevalent rheumatoid arthritis-associated interstitial lung disease (RA-ILD) using multicenter Chinese patient cohorts. We also evaluated the discriminative value of *MUC5B* polymorphisms (rs35705950 and rs868903) in a combined clinical-genetic model for this purpose.

**Methods:**

A multicenter, two-phase design was adopted. A total of 446 patients with rheumatoid arthritis (RA) from five medical centers were retrospectively enrolled in the discovery phase. Multivariate logistic regression was used to identify independent risk factors associated with RA-ILD and to construct a clinical risk identification model. A total of 238 patients with RA from three medical centers were then prospectively recruited for the validation phase and genotyped for *MUC5B* (rs35705950 and rs868903). External validation of the clinical model was performed first, followed by the construction of a combined clinical-genetic model. The model’s discrimination was assessed using receiver operating characteristic (ROC) curve analysis, and calibration was evaluated using the Hosmer–Lemeshow test.

**Results:**

In the discovery phase, smoking history (odds ratio [OR] = 2.00, P = 0.045), elevated rheumatoid factor (RF) (OR = 1.10 per 10 IU/mL, P = 0.037), a higher 28-joint disease activity score (DAS28) (OR = 1.20, P = 0.023), elevated serum carbohydrate antigen 19-9 (CA19-9) (OR = 3.70, P = 0.002), elevated lactate dehydrogenase (LDH) (OR = 1.13 per 10 U L, P < 0.001), and disease duration of ≥ 24 months (OR = 1.73, P = 0.029) were identified as independent risk factors for RA-ILD. Our clinical risk identification model yielded an area under the ROC curve (AUC) of 0.785 (95% confidence interval [CI]: 0.74–0.83). In the prospective validation cohort, we identified a novel association between *MUC5B* rs868903 and RA-ILD (OR = 2.58, P = 0.001), with both rs868903 and rs35705950 distinguishing patients with RA-ILD from those without ILD (P < 0.05).The combined model incorporating *MUC5B* genotypes achieved an AUC of 0.786, which did not differ significantly from that of the clinical model alone (P = 0.507). Although the genetic variants significantly improved continuous risk stratification (integrated discrimination improvement [IDI] = 0.020, P = 0.040; continuous net reclassification improvement [NRI] = 0.466, P < 0.001), categorical NRI at clinically relevant thresholds was non-significant.

**Conclusions:**

This multicenter study validated a clinical risk identification model for RA-ILD. We identified a novel association between *MUC5B* rs868903 and RA-ILD in a Chinese cohort. Although the incorporation of genetic information improved continuous risk estimation, it provided limited incremental value at standard clinical decision-making thresholds, indicating that clinical indicators play a dominant role in RA-ILD risk identification. The resultant clinical model may have broad potential for routine clinical use.

## 1 Introduction

Rheumatoid arthritis (RA) is a systemic autoimmune disease characterized by symmetrical polyarthritis and progressive joint destruction (1). Approximately 40–60% of patients with RA develop extra-articular manifestations, among which RA-associated interstitial lung disease (RA-ILD) represents the most severe and life-threatening pulmonary complication (2–5). In particular, RA-ILD typically has an insidious onset and heterogeneous clinical presentation, with a pooled clinical prevalence of 18.7%according to recent meta-analytic estimates (6). Over 90% of ILD cases are diagnosed after RA onset (mean interval 3.3 years) (7), although 10–20% of patients experience pulmonary symptoms that precede joint manifestations (4, 8). RA-ILD confers a substantially elevated mortality risk. Studies have shown a 3-fold higher mortality rate vs RA without ILD, with a 5-year survival rate of only 39–59.7% and a median survival of only 2.6–3.3 years following diagnosis (9–11). The identification of high-risk individuals thus represents an urgent unmet clinical need. Although male sex, smoking history, elevated rheumatoid factor (RF), and high the 28-joint disease activity score (DAS28) have been recognized as important risk factors in prior research, the evidence supporting these associations has mainly come from retrospective observational studies. Consistent, definitive conclusions have therefore not been fully established in this context (6, 12–14).

Beyond clinical parameters, genetic susceptibility plays a crucial role in the pathogenesis of RA-ILD. The rs35705950 variant of the *MUC5B* gene’s promoter represents the strongest known genetic risk factor for idiopathic pulmonary fibrosis (IPF) and upregulates MUC5B expression in the lung. However, its association with RA-ILD remains inconsistent across different patient populations (15–18).The rs868903 variant, located approximately 1.5 kb upstream of the *MUC5B* transcription start site, was identified as a significantly associated single nucleotide polymorphism (SNP) in the original Genome-Wide Association Study (GWAS) for idiopathic pulmonary fibrosis (IPF) and was subsequently validated in a Chinese IPF cohort as a risk factor for disease severity and survival (19, 20). However, this variant has not yet been investigated in patients with RA-ILD. According to the gnomAD v4 database, rs868903 is common in East Asians, with a C allele frequency of approximately 0.47. Furthermore, RegulomeDB v2 annotations indicate that this variant resides in a regulatory region with strong functional potential (score 1f, probability = 0.554), overlapping with eQTL evidence, transcription factor binding sites, and chromatin accessibility peaks. Although functional experimental evidence is currently lacking, the regulatory annotation, population frequency, and distinct position within the *MUC5B* promoter make rs868903 a biologically plausible candidate for modulating mucin expression in fibrotic lung disease. Its weak linkage disequilibrium with rs35705950 (r²= 0.0019, p = 0.373 based on 1000 Genomes East Asian data) suggests that these two loci can be analyzed independently. Whether adding genetic information to routine clinical and biochemical assessments meaningfully improves identification of prevalent RA-ILD risk remains unknown. Therefore, we conducted a multicenter, two-phase investigation to develop and externally validate a clinical risk identification model for prevalent RA-ILD. We also sought to systematically evaluate the added discriminative value of the rs35705950 and rs868903 *MUC5B* polymorphisms in this context.

## 2 Materials and methods

### 2.1 Study design and population

This multicenter, two-phase observational study was conducted at five tertiary medical centers in southeastern China. The study protocol was approved by the Ethics Committee of the Second Affiliated Hospital of Xiamen Medical College, and written informed consent was obtained from all participants.

#### 2.1.1 Discovery phase (retrospective cohort)

We retrospectively enrolled 446 patients from five participating centers between January 2018 to December 2022. Clinical and laboratory data were extracted from electronic medical records.

#### 2.1.2 Validation phase (prospective cohort)

We prospectively enrolled 238 patients from three independent centers between January 2023 to December 2025. In addition to clinical assessment, In addition to clinical evaluation, all participants underwent *MUC5B* genotyping for rs35705950 and rs868603 to assess genetic associations.

#### 2.1.3 Inclusion and exclusion criteria

Identical criteria were applied in both phases:

Inclusion criteria were: (1) age 18–75 years; (2) fulfillment of the 2010 ACR/EULAR classification criteria for RA (21); (3) available complete clinical and laboratory data. Exclusion criteria included: (1) concomitant primary pulmonary diseases (active infection, tuberculosis, COPD, bronchiectasis, lung malignancy); (2) interstitial lung disease attributable to non-RA etiologies (pneumoconiosis, radiation pneumonia); (3) history of malignancy; (4) pregnancy or lactation; (5) concurrent overlapping connective tissue diseases; (6) severe heart failure (NYHA class III–IV); (7) incomplete medical records with critical missing data.

RA-ILD was diagnosed when, in addition to meeting the above RA criteria, patients demonstrated ILD on chest high-resolution computed tomography (HRCT) with patterns compatible with the 2002 ATS/ERS classification of idiopathic interstitial pneumonias (22), with other known causes excluded. The specific HRCT criteria for each pattern were defined as follows: Usual interstitial pneumonia (UIP) was defined as bilateral, basal, and subpleural reticular abnormalities with honeycombing and/or traction bronchiectasis, without consolidation or nodules. Nonspecific interstitial pneumonia (NSIP) was defined as bilateral ground-glass and reticular opacities, predominantly subpleural and basal, with a symmetric distribution. Organizing pneumonia (OP) was defined as patchy bilateral consolidation, typically subpleural or peribronchial. Lymphoid interstitial pneumonia (LIP) was defined as diffuse reticular opacities and/or centrilobular nodules with ground glass attenuation. Unclassifiable was defined as patterns not meeting the above criteria. The images were independently evaluated in a blinded manner by two radiologists with more than 5 years of experience in thoracic imaging. Interobserver agreement was assessed using Cohen’s kappa statistic(κ= 0.78), indicating substantial agreement. Disagreements were resolved by consensus; in persistent cases, a third senior radiologist adjudicated the final diagnosis.

### 2.2 Demographic, clinical and laboratory data collection

Demographic and clinical data were collected, including age, sex, body mass index (BMI), smoking history (defined as ever vs. never), disease duration (months), and comorbidities such as hypertension, diabetes mellitus, coronary atherosclerotic cardiopathy, and carotid artery plaque. Laboratory assessments comprised complete blood count (white blood cell count, hemoglobin, platelet count), liver function tests (alanine aminotransferase, ALT; aspartate aminotransferase, AST; lactate dehydrogenase, LDH), renal function tests (blood urea nitrogen, BUN; creatinine, Cr), inflammatory markers (erythrocyte sedimentation rate, ESR; C-reactive protein, CRP), immunoglobulins (IgG, IgA, IgM), rheumatoid factor (RF), anti-cyclic citrullinated peptide (anti-CCP) antibodies, and tumor markers (carcinoembryonic antigen, CEA; carbohydrate antigen 19-9, CA19-9). Disease activity was assessed using the 28-joint Disease Activity Score (DAS28).

### 2.3 MUC5B polymorphism genotyping

Genomic DNA was extracted from 10 mL peripheral blood samples collected in EDTA-containing tubes using a commercial DNA extraction kit (Tiangen Biotech, Beijing, China) according to the manufacturer’s protocol. Genotyping for *MUC5B* rs35705950 (G>T) and rs868903 (T>C) was performed using polymerase chain reaction (PCR) followed by direct Sanger sequencing in the prospective validation cohort (n=238).For rs35705950, the primer sequences were: forward 5′-CCCCTTCCCGGCAGGAAC-3′ and reverse 5′-CTTTGCCCTCGTCCCTCC-3′. For rs868903, the primers were: forward 5′-TGCAACACCAGCTCACCAT-3′ and reverse 5′-TGTCCACAGCAGCATCACT-3′. PCR amplification was performed in a total volume of 50 μL containing: 1 μL forward primer (10 μM), 1 μL reverse primer (10 μM), 25 μL 2× Taq Master Mix, and 1 μL DNA template(Vazyme-innovation in enzyme technology, nanjing, China). Thermal cycling conditions consisted of initial denaturation at 94 °C for 5 minutes, followed by 30 cycles of 94 °C for 30 seconds, 60 °C for 30 seconds, and 72 °C for 30 seconds, with a final extension at 72 °C for 5 minutes. PCR products were purified and sequenced by Xiamen Borui Biotechnology using an ABI 3730xl DNA Analyzer (Applied Biosystems, USA). Genotypes were determined by two independent investigators blinded to clinical status. Hardy-Weinberg equilibrium was assessed using the χ² test.

### 2.4 Statistical methods

Statistical analyses were performed using R version 4.5.2 (R Foundation for Statistical Computing, Vienna, Austria) and IBM SPSS Statistics 23.0 (Armonk, NY). The Kolmogorov–Smirnov test evaluated normality assumptions for continuous variables. Data following normal distribution are presented as mean ± standard deviation (SD), while non-normally distributed data are expressed as median (interquartile range, IQR). Categorical variables are reported as frequencies and percentages. Between-group comparisons utilized independent t-tests or Mann–Whitney U-tests for continuous data, and χ² or Fisher’s exact tests for categorical data, as appropriate. Univariate logistic regression identified potential predictors of RA-ILD (P < 0.05). Significant variables were subsequently entered into a multivariate logistic regression model employing backward stepwise selection to determine independent risk factors. Discriminative ability was assessed by the area under the receiver operating characteristic curve (AUC), calculated using the “pROC” package (v1.19.0.1). Model calibration was formally evaluated by the Hosme–Lemeshow goodness-of-fit test (“ResourceSelection” package, v0.3-6). The calibration curve was plotted with bootstrap bias correction (1,000 resamples via the “calibrate” function in the “rms” package, v8.1) to adjust for overfitting. The DeLong test compared AUCs between models. Multicollinearity was assessed using the variance inflation factor (VIF). Bootstrap validation with 1,000 resamples was performed to assess the optimism-corrected AUC and variable selection frequencies. Additionally, clinically forced variables and LASSO logistic regression with 10-fold cross-validation were performed as a sensitivity analysis to confirm the robustness of variable selection. Genetic association analysis was performed in the prospective cohort (n=238). Hardy–Weinberg equilibrium was assessed using Pearson’s χ² test. Genotype distributions (dominant model: wild-type homozygote vs. variant carriers) and allele frequencies were compared between RA-ILD and RA-only groups using χ² or Fisher’s exact tests. Odds ratios (OR) and 95% CIs were calculated for the association between *MUC5B* variants and RA-ILD risk. *Post-hoc* power analysis was performed using the “pwr” package. Net reclassification improvement (NRI) and integrated discrimination improvement (IDI) were calculated to quantify the incremental predictive value of genetic information. Decision curve analysis (DCA) was conducted to evaluate net clinical benefit across various threshold probabilities. A two-tailed P value < 0.05 was considered statistically significant.

## 3 Results

### 3.1 Baseline characteristics of the discovery cohort

A total of 446 patients with RA were enrolled in the retrospective discovery phase, comprising 150 patients with ILD (33.6%) and 296 without ILD (66.4%), with a sampling ratio of 1:2. The baseline demographic, clinical, and laboratory characteristics are summarized in **Table 1**. Compared to RA patients without ILD, those with RA-ILD were significantly older (mean age 62.8 ± 10.1 vs. 55.7 ± 12.2 years, P = 0.016) and had a higher male proportion (34.7% vs. 24.0%, P = 0.019). Disease duration was significantly longer in the RA-ILD group (median 91.3, IQR 76.6–106.1 vs. 75.7, IQR 64.7–86.6 months, P = 0.020). Smoking history was significantly more prevalent among RA-ILD patients (20.0% vs. 7.1%, P < 0.001). Regarding comorbidities, the prevalence of coronary atherosclerotic cardiopathy was significantly higher in the RA-ILD group (6.7% vs. 2.4%, P = 0.035), as was the prevalence of carotid artery plaque (55.3% vs. 44.6%, P = 0.035). No significant differences were observed in BMI, hypertension, or diabetes mellitus between the two groups (all P > 0.05). RA-ILD patients exhibited significantly higher disease activity, as shown by elevated DAS28 scores (mean 4.9 ± 1.6 vs. 4.5 ± 1.4, P = 0.001). Serological markers of RA severity were markedly increased in the ILD group, including RF (median 258.0, IQR 241.2–301.8 vs. 168.5, IQR 142.9–194.2 IU/mL, P = 0.001) and anti-CCP antibodies (median 214.5, IQR 183.6–245.3 vs. 165.2, IQR 144.6–185.9 IU/mL, P < 0.001). Inflammatory markers were also significantly elevated, with higher ESR (median 62.3, IQR 56.7–68.1 vs. 49.7, IQR 46.3–55.5 mm/h, P < 0.001) and CRP (median 33.0, IQR 26.6–39.2 vs. 22.1, IQR 19.0–25.1 mg/L, P = 0.001) in the RA-ILD cohort. LDH levels were higher in the RA-ILD group (mean 203.6 ± 82.4 vs. 145.2 ± 72.8 U/L, P < 0.001). The proportion of patients with elevated CEA was significantly higher in the RA-ILD group (10.7% vs. 2.6%, P = 0.001), as was the proportion with elevated CA19-9 (12.7% vs. 4.2%, P < 0.001), which suggests the potential involvement of these markers in RA-ILD pathophysiology.

**Table 1:** Demographic, clinical features and laboratory measures of rheumatoid arthritis (RA) patients with or without interstitial lung disease (ILD).

Characteristic	Without ILD involvement (N = 296)	With ILD involvement (N = 150)	*P* value
Sex, male n(%)	71 (24.0)	52 (34.7)	0.019
age (mean ± SD), years	55.7 ± 12.2	62.8 ± 10.1	0.016
Disease duration, months	75.7 (64.71,86.6)	91.3 (76.56,106.1)	0.020
BMI	22.2 (21.9,22.6)	22.6 (21.9,23.2)	0.335
smoking history n(%)	21 (7.1)	30 (20.0)	< 0.001
hypertension n(%)	73 (24.7)	47 (31.3)	0.143
diabetes n(%)	21 (7.1)	18 (12.0)	0.109
Coronary atherosclerotic cardiopathy n(%)	7 (2.4)	10 (6.7)	0.035
Carotid artery plaque n(%)	132 (44.6)	83(55.3)	0.035
RF IU/mL	168.5 (142.9,194.2)	258 (241.2,301.8)	0.001
Anti-CCP antibodies IU/mL	165.2 (144.6,185.9)	214.5 (183.6,245.3)	< 0.001
ESR, mm/h	49.7 (46.3,55.5)	62.3 (56.7,68.1)	< 0.001
CRP, mg/L	22.1 (19.0,25.1)	33.0 (26.6,39.2)	0.001
DAS 28	4.5 ± 1.4	4.9 ± 1.6	0.001
WBC(mean ± SD)10^9^/L	7.1 ± 2.3	7.5 ± 2.9	0.079
Hb(mean ± SD)g/L	118.8 ± 19.3	119.2 ± 27.1	0.850
PLT(mean ± SD)10^9^/L	278.7 ± 104.8	272.4 ± 102.70	0.545
ALT(mean ± SD)u/L	22.7 ± 21.6	21.0 ± 19.7	0.392
AST(mean ± SD)u/L	22.4 ± 13.9	25.5 ± 20.8	0.061
LDH(mean ± SD)u/L	145.2 ± 72.8	203.6 ± 82.4	< 0.001
BUN mmol/L	5.3 (5.09,5.99)	5.6 (5.2,6.1)	0.257
Cr umol/L	56.5 (54.6,58.5)	63.0 (58.2,68.0)	0.452
IgG(mean ± SD)mg/mL	15.8 ± 4.5	17.5 ± 7.4	0.148
IgA(mean ± SD)mg/mL	2.9 ± 1.8	3.0 ± 2.3	0.687
IgM(mean ± SD)mg/mL	1.4 ± 0.8	1.5 ± 1.0	0.737
CEA> 5ng/L n(%)	8 (2.6)	16 (10.7)	0.001
CA199> 35ng/L n(%)	7 (2.4)	19 (12.7)	< 0.001

BMI, Standard Body Mass Index; RF, rheumatoid factor; CCP, anti-cyclic citrullinated peptide; ESR, erythrocyte sedimentation rate; CRP, C-reactive protein; DAS, Disease Activity Score; WBC, white blood cell; Hb, hemoglobin; PLT Platelet; ALT, alanine transaminase; AST, aspartate aminotransferase; LDH, lactate dehydrogenase; BUN, Blood usea nitrogen; Cr, Creatinine; IgA, immunoglobulin A; IgG, immunoglobulin G; IgM, immunoglobulin M; CEA, carcinoembryonic antigen; CA199, Carbohydrate antigen 19-9.

### 3.2 Identification of independent risk factors with ILD involvement in RA

In the discovery phase, univariate logistic regression analysis identified multiple candidate risk factors associated with RA-ILD (P < 0.05), including male sex, age >65 years, disease duration ≥ 24months, smoking history, coronary atherosclerotic cardiopathy, carotid artery plaque, RF, anti-CCP antibodies, ESR, DAS28 score, LDH, elevated CEA, and elevated CA19-9 (**Table 2**). Subsequent multivariate logistic regression analysis identified six independent predictors of RA-ILD (**Table 2**). Disease duration ≥ 24months remained significantly associated with ILD (adjusted OR = 1.73, 95%CI: 1.07–2.86, P = 0.029). Smoking history demonstrated a two-fold increased risk (adjusted OR = 2.00, 95%CI: 1.01–3.96, P = 0.045). Higher RF levels (adjusted OR = 1.10 per 10 IU/mL, 95% CI: 1.02–1.19, P = 0.037) and elevated DAS28 scores (adjusted OR = 1.20 per unit, 95%CI: 1.03–1.41, P = 0.023) were independent risk factors. Higher LDH levels (adjusted OR = 1.13 per 10 U/L, 95% CI: 1.08–1.20, P<0.001) and elevated CA19-9 (adjusted OR = 3.70, 95%CI: 1.43–10.4, P = 0.009) were also independently associated with ILD. Other factors did not retain significance after adjustment.

**Table 2:** Univariate and stepwise multivariate analysis of risk factors for ILD involvement in rheumatoid arthritis.

Characteristics	Univariate analysis			Multivariate analysis		
	Odds ratios	95% CI	*P* value	Odds ratios	95% CI	*P* value
Sex, male n (%)	1.68	1.10-2.58	0.018	–		
Age(65) years	2.50	1.63-3.80	< 0.001	–		
Disease duration, (24) months	1.71	1.12-2.67	0.015	1.73	1.07-2.86	0.029
smoking history n (%)	3.41	1.89-6.25	0.001	2.00	1.01-3.96	0.045
Coronary atherosclerotic cardiopathy n (%)	2.95	1.11-8.28	0.031	–		
Carotid artery plaque n (%)	1.54	1.04-2.30	0.032	–		
RF per10 IU/mL	1.10	1.02-1.20	0.001	1.10	1.02-1.19	0.037
Anti-CCP antibodies	1.01	1.01-1.02	0.008	–		
ESR, mm/h	1.01	1.00-1.01	< 0.001	–		
CRP, mg/L	1.01	1.00-1.02	0.225	–		
DAS 28	1.25	1.09-1.43	< 0.001	1.20	1.03-1.41	0.023
LDH per 10 u/L	1.12	1.07-1.17	< 0.001	1.13	1.08-1.20	< 0.001
CEA> 5ng/L n(%)	1.53	1.27-1.91	< 0.001	–		
CA199> 35ng/L n(%)	5.99	2.56-15.65	0.001	3.70	1.43-10.4	0.009

### 3.3 Establishment and evaluation of a risk identification model for RA-ILD

**Figure 1:**
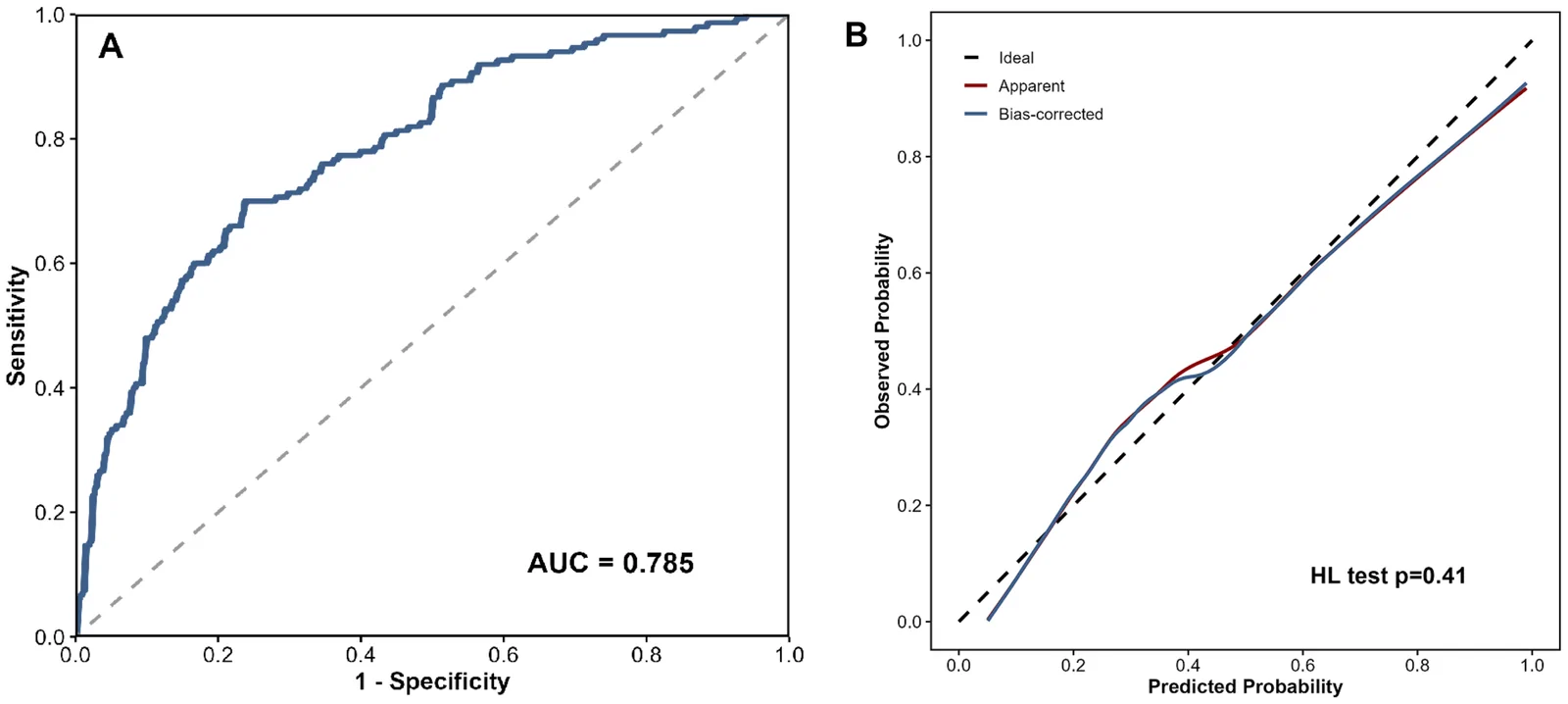
Evaluation of the performance of the risk identification model. **(A)** Receiver operating characteristic (ROC) curve of the identification model, the AUC was 0.785. **(B)** Calibration curve for the logistic regression model. The calibration curve was plotted with bootstrap bias correction (1,000 resamples) to adjust for overfitting. p-value was calculated with Hosmer–Lemeshow (HL) goodness-of-fit test(p=0.41). The solid blue line represents orginal calibration curve, Blue bands represent bias-corrected calibration curve.

Using the six independent predictors identified (disease duration ≥ 24 months, smoking history, RF, DAS28, LDH, and elevated CA19-9), we constructed a clinical identification model based on multivariate logistic regression coefficients. The model showed good discrimination in the discovery cohort, with an area under the ROC curve (AUC) of 0.785 (95%CI: 0.74–0.83) (**Figure 1A**). Calibration assessment using the Hosmer-Lemeshow goodness-of-fit test indicated excellent agreement between predicted probabilities and observed outcomes (χ² = 6.3, P = 0.41). The calibration plot showed close concordance between the bias-corrected curve and the ideal diagonal across the range of predicted probabilities (**Figure 1B**). Multicollinearity assessment revealed variance inflation factor (VIF) values < 2 for all retained variables (range: 1.06–1.80), indicating no significant multicollinearity. Bootstrap validation yielded an optimism-corrected AUC of 0.773 (95% CI: 0.723–0.812), closely approximating the apparent AUC (0.785) (**Supplementary Figure 1**). The six retained variables showed high selection frequencies (68%–100%) across 1000 bootstrap resamples, confirming their stability. The clinically forced model and LASSO logistic regression with 10-fold cross-validation were performed as sensitivity analyses. The optimal lambda selected six variables, which were identical to those in our final stepwise model (smoking, RF, DAS28, LDH, CA19-9, and disease duration ≥24 months).These consistent results across different analytical strategies support the robustness of the final six-variable model (**Supplementary Figure 2**).

### 3.4 *MUC5B* polymorphisms and RA-ILD risk in the prospective cohort

All 238 patients in the prospective validation cohort underwent successful genotyping. Both single nucleotide polymorphisms (SNPs) were in Hardy-Weinberg equilibrium in the overall cohort (rs35705950: P = 0.54; rs868903: p= 0.20) (P > 0.05).

#### 3.4.1 rs35705950 (G>T) variant

Genotype distributions differed between RA patients with and without ILD. The risk genotype (GT or TT) was present in 16.3% (13/80) of RA-ILD patients versus 6.3% (10/158) of those without ILD (OR = 2.87, 95% CI: 1.20–6.88, P = 0.026). At the allelic level, the T allele was more frequent in the RA-ILD group (8.8% vs. 3.1%, OR = 2.93, 95% CI: 1.27–6.76, P = 0.016) (**Table 3**).

**Table 3:** Genotypic association of *MUC5B* rs35705950 and rs868903 single-nucleotide polymorphisms (SNPs) in RA patients with and without ILD.

	RA(n=158) n(%)	RA-ILD(n=80) n(%)	Odds ratios	95% CI	*P* value
rs35705950 G>T Genotype					
GG	148 (93.7)	67 (83.8)	–		
GT+TT	10 (6.3)	13 (16.3)	2.87	1.20-6.88	0.026
rs35705950 G>T Allele					
G	306 (96.8)	146 (96.2)	–		
T	10 (3.1)	14 (8.8)	2.93	1.27- 6.76	0.016
rs868903 T>C Genotype					
TT	98 (62.0)	31 (38.8)	–	–	–
TC+CC	60 (38.0)	49 (61.3)	2.58	1.49-4.49	0.001
rs868903 T>C Allele					
T	246 (77.8)	99 (61.9)	–		
C	70 (22.2)	61 (38.1)	2.17	1.43-3.28	< 0.001

#### 3.4.2 rs868903 (T>C) variant

The rs868903 polymorphism exhibited a more robust relationship with RA-ILD. The TC/CC genotype was identified in 61.3 % (49/80) of ILD cases versus 38.0 % (60/158) of controls (OR = 2.58, 95 % CI 1.49–4.49, P = 0.001). Moreover, the C-allele frequency was higher among ILD patients (38.1% vs.22.2%;OR=2.17,95%CI 1.43-3.28, P < 0.001) (**Table 3**).

#### 3.4.3 Linkage disequilibrium analysis

Linkage disequilibrium between rs868903 and rs35705950 was weak (r^2^ = 0.01, D’=0.47), indicating that these two variants are inherited independently and can be analyzed as separate loci.

#### 3.4.4 *Post-hoc* power analysis

Power calculations were performed based on the observed effect sizes and allele frequencies α = 0.05 (two-tailed). For rs868903, the study had 91.9% power to detect an OR of 2.58. For rs35705950, power was 66.8% to detect an OR of 2.87, which suggests relatively limited statistical power for this low-frequency variant.

### 3.5 External validation and model performance

#### 3.5.1 Nomogram development

**Figure 2:**
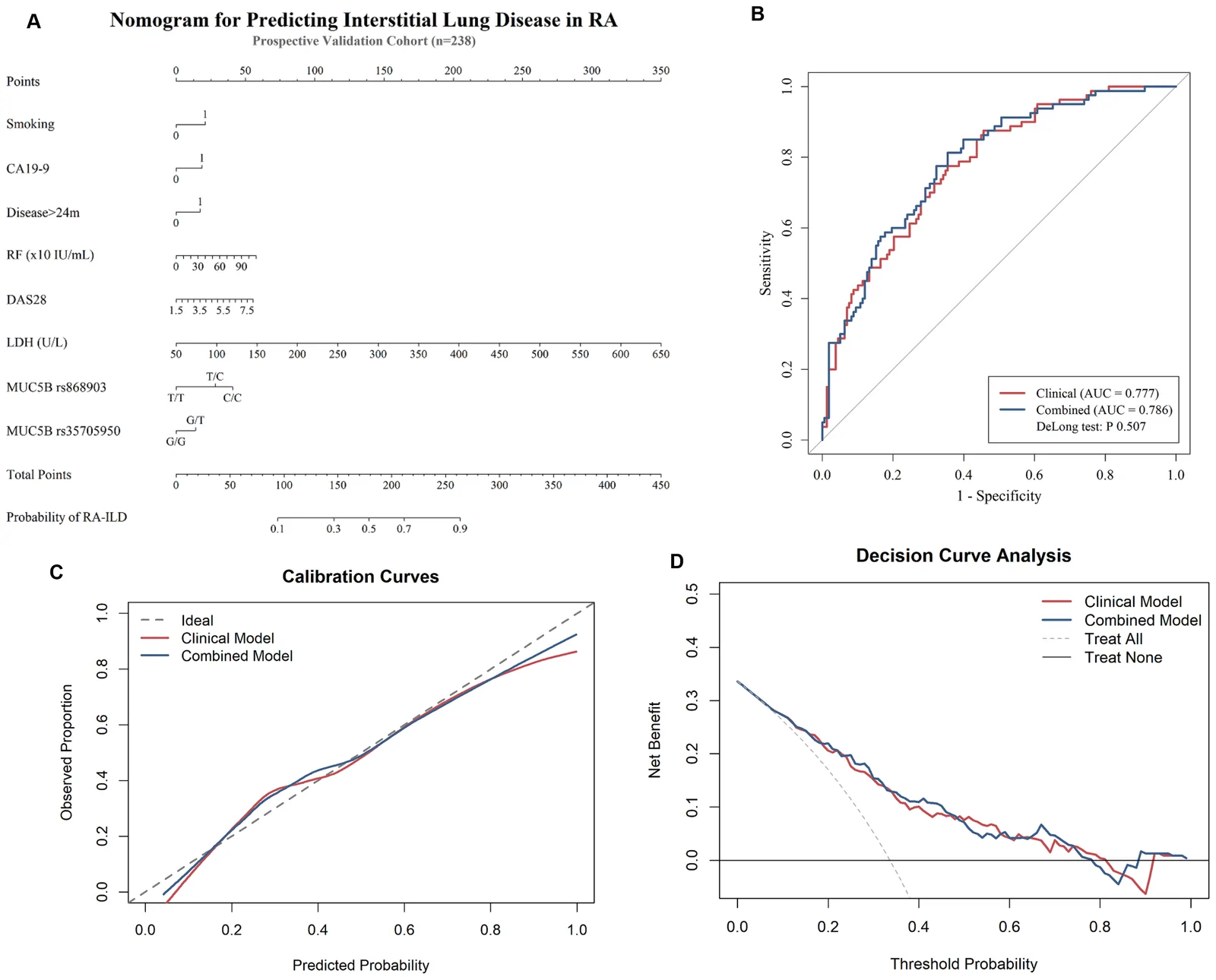
Development and validation of the clinical identification model for RA-ILD in the prospective cohort. **(A)** Nomogram incorporating six clinical variables and two MUC5B genetic variants. **(B)** ROC curves comparing the clinical model (red) and combined model (blue). **(C)** Calibration curves for both models against the ideal line (dashed). **(D)** Decision curve analysis showing net benefit across threshold probabilities; both models outperformed “treat all” (dashed gray) and were nearly superimposed.

We developed a nomogram that integrates the six clinical predictors (smoking history, CA19–9 elevation, disease duration ≥ 24 months, RF levels, DAS28 score, and LDH levels) and the two *MUC5B* variants (rs35705950 and rs868903) (**Figure 2A**). Total scores range from 0 to 450 and map to predicted probabilities of RA-ILD between 0.1 and 0.9.

#### 3.5.2 Discriminative performance

In the prospective validation cohort (n = 238), the clinical model achieved an AUC of 0.777 (95% CI: 0.71–0.84). The combined model achieved an AUC of 0.786 (95% CI: 0.72–0.85), but the incremental improvement was not statistically significant (ΔAUC = 0.009, P = 0.507) (**Figure 2B**).

#### 3.5.3 Model calibration

Both models were well calibrated across the spectrum of predicted risk (**Figure 2C**). The Hosmer–Lemeshow test supported adequate fit for the clinical model (χ² = 9.8, P = 0.27) and combined model (χ² = 3.3, P = 0.91).

#### 3.5.4 Reclassification and incremental value

We further assessed whether *MUC5B* genotyping improved risk reclassification using the NRI and IDI. The combined model demonstrated significant improvement in integrated discrimination (IDI = 0.020, 95% CI: 0.001–0.039, P = 0.040) and continuous risk estimation (continuous NRI = 0.466, 95% CI: 0.204–0.727, P < 0.001). However, at clinically relevant categorical thresholds (0.3, 0.4, 0.5, and 0.7), NRI estimates were non-significant (range: −0.007 to 0.044, all P > 0.05). indicating that while MUC5B genotyping refines continuous risk estimation, it does not improve classification at standard clinical decision-making thresholds.

#### 3.5.5 Clinical utility

Decision curve analysis demonstrated that both the clinical and combined models provided positive net benefit compared with the “treat none” strategy across clinically relevant threshold probabilities (10–70%), with equivalent performance between the two models (**Figure 2D**). The two curves remained virtually superimposed across all threshold probabilities, indicating that while *MUC5B* genotyping refines continuous risk estimates, it does not appreciably alter clinical treatment decisions at standard risk thresholds.

## 4 Discussion

RA is a chronic systemic autoimmune disease characterized by aberrant immune activation, synovial hyperplasia, and progressive joint destruction (1, 2). Recent studies have demonstrated that dysregulated osteoclast differentiation and ferroptosis-mediated synovial cell death contribute to RA pathogenesis, underscoring the multifaceted pathophysiology of RA and the need for validated risk identification tools for its severe complications (23, 24). ILD is among the most severe pulmonary manifestations of RA, being associated with poor prognosis and higher mortality (14, 25, 26). The early identification of high-risk patients and the use of validated risk assessment tools are therefore essential for guiding clinical management. In this multicenter, two-phase study, we developed and prospectively validated a clinical identification model for RA-ILD that incorporated six variables: smoking history, elevated CA19–9 levels, disease duration of ≥ 24 months, RF level, DAS28 score, and LDH level. The model demonstrated good discriminative performance and excellent calibration, comparable to other similar tools that have been reported recently, in both our development and prospective validation cohorts. For instance, the clinical–genetic model developed by Juge et al. using European patient populations reported an AUC of 0.82 (27), whereas validation studies in a Korean cohort demonstrated an AUC of 0.78 (28). However, the Juge model required multi-locus genetic testing, and the KORAIL model relied on baseline pulmonary function tests and longitudinal HRCT follow-up, both of which add substantial cost and complexity to risk assessment. Our model achieved similar identification efficacy using only routinely available clinical and biochemical indicators, thus potentially offering greater accessibility and practicality in resource-limited settings.

Our findings regarding clinical risk factors align closely with the established literature. Smoking history emerged as an independent risk factor, consistent with prior studies that identified tobacco exposure as the strongest environmental risk factor, with effect sizes ranging from 1.9- to 8.8-fold across cohorts (6, 14). Higher DAS28 scores were similarly associated with ILD risk, supporting the notion that active systemic inflammation extends to the lungs. Prior studies have also linked moderate or high disease activity to substantially increased risks (6, 29, 30). High-titer auto-antibodies correlate with severe, systemic RA phenotypes that are prone to extra-articular manifestations, as was demonstrated in recent meta-analyses (6, 29, 31).

Our study also found that a disease duration of ≥ 24 months significantly increased the risk of ILD. Li et al. similarly identified short RA duration (0–5 years) as an independent risk factor for ILD in their retrospective analysis, which is consistent with our observation that the risk increases after just 2 years of disease (13). Hyldgaard et al. found that 34.0% of RA-ILD cases were diagnosed within 1 year before or after RA diagnosis, with the mortality risk peaking over the first months following ILD detection (9). In a large-scale analysis of 8,963 real-world patients with RA, Zhuo et al. reported that 91.8% of RA-ILD cases were diagnosed after RA onset, with a mean interval of only 3.3 years between the two (7). Taken together, these data indicate that the window of highest susceptibility to ILD may be concentrated over the first 2–5 years after RA onset. This potentially challenges the more traditional view of ILD as a late complication of long-standing RA.

This study also identified elevated serum CA19–9 and LDH levels as novel risk factors for ILD. CA19–9 has emerged as a strong independent risk factor associated with RA-ILD, as elevated levels reflect active epithelial injury and remodeling in fibrotic lung disease (32–34). CA19–9 and CEA are established markers of epithelial damage, and their elevation in ILD is attributed to the regeneration of damaged lung epithelium. Specifically, CA19–9 is produced by bronchial gland cells, which proliferate and secrete it in response to chronic pulmonary fibrosis; inflammatory cells can further contribute to its production, linking elevated CA19–9 to the active inflammatory phase of ILD (35). However, our data show that the proportion of patients with elevated CA19–9 was relatively low(12.7% in the ILD group vs. 2.4% in the non-ILD group). CA19–9 elevation may reflect epithelial injury in ILD but is non-specific, as it can also be elevated in pancreatic malignancy, biliary obstruction, and other gastrointestinal conditions. Therefore, clinicians should exclude these alternative etiologies through appropriate imaging and clinical evaluation before attributing CA19–9 elevation to ILD-related epithelial injury, thereby avoiding misdiagnosis. Elevated LDH levels have also emerged as a significant biochemical factor, consistent with recent retrospective data from China suggesting that they increase the risk of RA-ILD (36). AST levels showed a trend toward elevation in the RA-ILD group (P = 0.061), but their broad tissue distribution across the liver, skeletal muscle, and other organs confers lower specificity for interstitial lung injury compared with LDH, thereby excluding AST from the final model. These readily available biochemical parameters offer cost-effective alternatives to specialized biomarkers, and may facilitate risk stratification in resource-limited settings.

We also identified significant associations of the rs35705950 and rs868903 variants in the *MUC5B* promoter with RA-ILD risk in our Chinese patient population. Although the association between the rs35705950 variant and RA-ILD has been previously established in European, American, and Korean cohorts, this variant is extremely rare in East Asian populations, which markedly limits its detectability as a risk locus in Asian cohorts (15–18), In contrast, this study identified a novel association for the rs868903 in a Chinese patient cohort. Seibold et al. originally identified rs868903 as one of five significantly associated SNPs with IPF in the MUC5B promoter region, and Wang et al. subsequently validated its association with IPF severity and survival in a Chinese cohort (19, 20). RegulomeDB v2 annotations indicate that rs868903 resides in a region with high regulatory potential, suggesting that it may modulate baseline *MUC5B* promoter activity. The weak linkage disequilibrium between rs868903 and rs35705950 in our cohort (r² = 0.01, D’ = 0.47), consistent with 1000 Genomes East Asian data, further supports an independent genetic effect, suggesting that these variants may modulate gene expression through distinct mechanisms. Future studies should employ reporter gene assays, electrophoretic mobility shift assays, and chromatin immunoprecipitation to determine whether the C allele of rs868903 alters transcription factor binding and MUC5B expression in lung epithelial cells. For rs868903, our study achieved 91.9% power to detect an OR of 2.58, supporting the robustness of this novel association. For rs35705950, statistical power was limited to 66.8% due to the low-frequency variant; therefore, the observed association (OR = 2.87) should be interpreted with caution and warrants validation in larger cohorts. Despite the significant associations observed for both rs868903 and rs35705950, the incorporation of these variants into our predictive model only produced a minimal increase in AUC, without any significant improvement in categorical classification. Our restricted two-SNP panel cannot capture the full genetic architecture of RA-ILD risk, whereas polygenic risk scores integrating multiple loci may offer greater predictive value.

The validated nomogram developed in this study offers an immediate bedside tool for personalized ILD risk stratification that may help clinicians identify high-risk patients who merit further evaluations using HRCT, as well as avoid unnecessary imaging in lower-risk patients. Given the modest incremental value of the two-SNP panel of *MUC5B* genotyping that we observed, we recommend that routine RA-ILD screening rely primarily on the clinical–biochemical model. However, genetic testing for *MUC5B* variants, particularly rs868903, may serve as a refinement tool for patients with intermediate clinical risk profiles from whom management decisions remain equivocal. Future investigations should consider focusing on the development of comprehensive polygenic risk scores and prospectively validating such expanded panels in patient cohorts with incident RA, in order to determine whether precision genetic profiling can provide clinically meaningful improvements in terms of predicting ILD risk.

This study was subject to several key limitations that merit consideration. First, this is a cross-sectional risk identification model distinguishing RA patients with prevalent ILD from those without ILD at the time of assessment, rather than a longitudinal prediction model for incident ILD. Therefore, future prospective longitudinal studies tracking RA patients are needed to validate whether these clinical and genetic markers predict incident ILD development. Second, although our validation cohort employed a prospective design, the modest sample size (n = 238) and limited number of SNPs tested (n = 2) constrained our ability to detect the full potential value of genetic information and precluded subgroup analyses, particularly regarding UIP versus NSIP pattern prediction. Future studies should evaluate whether genetic variants differentially predict specific histopathological phenotypes. Third, our study included patients with prevalent rather than incident ILD, thus limiting our model’s ability to predict new-onset disease in patients with early-stage RA. Fourth, although we identified CA19–9 and LDH as novel predictors, the exact pathophysiological mechanisms linking these markers to the development of RA-ILD merit further elucidation.

This multicenter, prospective validation study established an identification model for RA-ILD risk based on routine clinical and biochemical indicators, and reported a novel association for the rs868903 genetic variant of the *MUC5B* gene promoter in this context, in a Chinese patient cohort. Although genetic variants are significantly associated with disease risk, current two-SNP testing offers limited incremental value for clinical decision-making. This suggests that more comprehensive genetic profiling will be required to achieve more precise prediction of ILD risk.

## Data Availability

Data used in this study are available from the first author upon reasonable request.

## References

[1] Di Matteo A Bathon JM Emery P . Rheumatoid arthritis. Lancet (London England). (2023) 402:2019–33. doi: 10.1016/S0140-6736(23)01525-8 38240831

[2] Kadura S Raghu G . Rheumatoid arthritis-interstitial lung disease: manifestations and current concepts in pathogenesis and management. Eur Respir Rev. (2021) 30(160):210011. doi: 10.1183/16000617.0011-2021 34168062 PMC9489133

[3] Turesson C O'Fallon WM Crowson CS Gabriel SE Matteson EL . Extra-articular disease manifestations in rheumatoid arthritis: incidence trends and risk factors over 46 years. Ann Rheumatic Dis. (2003) 62:722–7. doi: 10.1136/ard.62.8.722 12860726 PMC1754626

[4] Shao Y Zhang H Shi Q Wang Y Liang Q . Clinical prediction models of rheumatoid arthritis and its complications: focus on cardiovascular disease and interstitial lung disease. Arthritis Res Ther. (2023) 25:159. doi: 10.1186/s13075-023-03140-5 37658422 PMC10472585

[5] Manfredi A Cassone G Luppi F Atienza-Mateo B Cavazza A Sverzellati N . Rheumatoid arthritis related interstitial lung disease. Expert Rev Clin Immunol. (2021) 17:485–97. doi: 10.1080/1744666x.2021.1905524 33779447

[6] Wang HF Wang YY Li ZY He PJ Liu S Li QS . The prevalence and risk factors of rheumatoid arthritis-associated interstitial lung disease: a systematic review and meta-analysis. Ann Med. (2024) 56:2332406. doi: 10.1080/07853890.2024.2332406 38547537 PMC10984230

[7] Zhuo J Lama S Knapp K Gutierrez C Lovett K Thai S . Epidemiology and clinical characteristics of interstitial lung disease in patients with rheumatoid arthritis from the JointMan database. Sci Rep. (2023) 13:11678. doi: 10.1038/s41598-023-37452-y 37468565 PMC10356939

[8] Alunno A Gerli R Giacomelli R Carubbi F . Clinical, epidemiological, and histopathological features of respiratory involvement in rheumatoid arthritis. BioMed Res Int. (2017) 2017:7915340. doi: 10.1155/2017/7915340 29238722 PMC5697381

[9] Hyldgaard C Hilberg O Pedersen AB Ulrichsen SP Løkke A Bendstrup E . A population-based cohort study of rheumatoid arthritis-associated interstitial lung disease: comorbidity and mortality. Ann Rheumatic Dis. (2017) 76:1700–6. doi: 10.1136/annrheumdis-2017-211138 28611082

[10] Zamora-Legoff JA Krause ML Crowson CS Ryu JH Matteson EL . Patterns of interstitial lung disease and mortality in rheumatoid arthritis. Rheumatol (Oxford England). (2017) 56:344–50. doi: 10.1093/rheumatology/kew391 27940586 PMC6251572

[11] Bongartz T Nannini C Medina-Velasquez YF Achenbach SJ Crowson CS Ryu JH . Incidence and mortality of interstitial lung disease in rheumatoid arthritis: a population-based study. Arthritis Rheumatism. (2010) 62:1583–91. doi: 10.1002/art.27405 20155830 PMC4028137

[12] Fazeli MS Khaychuk V Wittstock K Han X Crocket G Lin M . Rheumatoid arthritis-associated interstitial lung disease: epidemiology, risk/prognostic factors, and treatment landscape. Clin Exp Rheumatol. (2021) 39:1108–18. doi: 10.55563/clinexprheumatol/h9tc57 33635222

[13] Li L Liu R Zhang Y Zhou J Li Y Xu Y . A retrospective study on the predictive implications of clinical characteristics and therapeutic management in patients with rheumatoid arthritis-associated interstitial lung disease. Clin Rheumatol. (2020) 39:1457–70. doi: 10.1007/s10067-019-04846-1 31858341

[14] Namas RA El-Kaissi M Memisoglu E Khan S Al Qassimi SS Omair MA . Rheumatoid arthritis-associated interstitial lung disease: clinical characteristics and mortality in a large single-centre cohort. Clin Exp Rheumatol. (2025) 43:425–34. doi: 10.55563/clinexprheumatol/d8m0xv 39932782

[15] Juge PA Lee JS Ebstein E Furukawa H Dobrinskikh E Gazal S . MUC5B promoter variant and rheumatoid arthritis with interstitial lung disease. N Engl J Med. (2018) 379:2209–19. doi: 10.1056/NEJMoa1801562 30345907 PMC6371965

[16] Wang N Zhang Q Jing X Guo J Huang H Xu Z . The association between MUC5B mutations and clinical outcome in patients with rheumatoid arthritis-associated interstitial lung disease: a retrospective exploratory study in China. Med Sci Monitor Int Med J Exp Clin Res. (2020) 26:e920137. doi: 10.12659/msm.920137 32142504 PMC7077059

[17] Juge PA Solomon JJ van Moorsel CHM Garofoli R Lee JS Louis-Sydney F . MUC5B promoter variant rs35705950 and rheumatoid arthritis associated interstitial lung disease survival and progression. Semin Arthritis Rheumatism. (2021) 51:996–1004. doi: 10.1016/j.semarthrit.2021.07.002 34411838

[18] Palomäki A Palotie A Koskela J Eklund KK Pirinen M Ripatti S . Lifetime risk of rheumatoid arthritis-associated interstitial lung disease in MUC5B mutation carriers. Ann Rheumatic Dis. (2021) 80:1530–6. doi: 10.1136/annrheumdis-2021-220698 PMC8600604 34344703

[19] Seibold MA Wise AL Speer MC Steele MP Brown KK Loyd JE . A common MUC5B promoter polymorphism and pulmonary fibrosis. N Engl J Med. (2011) 364:1503–12. doi: 10.1056/nejmoa1013660 21506741 PMC3379886

[20] Wang H Zhuang Y Peng H Cao M Li Y Xu Q . The relationship between MUC5B promoter, TERT polymorphisms and telomere lengths with radiographic extent and survival in a Chinese IPF cohort. Sci Rep. (2019) 9:15307. doi: 10.1038/s41598-019-51902-6 31653936 PMC6814782

[21] Neogi T Aletaha D Silman AJ Naden RL Felson DT Aggarwal R . The 2010 American College of Rheumatology/European League Against Rheumatism classification criteria for rheumatoid arthritis: phase 2 methodological report. Arthritis Rheumatism. (2010) 62:2582–91. doi: 10.1002/art.27580 20872596 PMC3077961

[22] American Thoracic Society/European Respiratory Society International Multidisciplinary Consensus Classification of the Idiopathic Interstitial Pneumonias . This joint statement of the American Thoracic Society (ATS), and the European Respiratory Society (ERS) was adopted by the ATS board of directors, June 2001 and by the ERS Executive Committee, June 2001. Am J Respir Crit Care Med. (2002) 165:277–304. doi: 10.1164/ajrccm.165.2.ats01 11790668

[23] Cao J Shen X Chen Q Wu J Yin Y Zhou E . Coumestrol induces ferroptosis to alleviate proliferation and inflammation responses of fibroblast-like synoviocytes in rheumatoid arthritis by inhibiting TRIM3-mediated down-regulation of mitochondrial PMAIP1. Lett Drug Design Discov. (2025) 22:100284. doi: 10.1016/j.lddd.2026.100284 42574925

[24] Cui J Li J Liu M Deng Y Xian J Ma J . Investigating the therapeutic potential of jiawei xuanbi decoction in rheumatoid arthritis via network pharmacology and cell experiments. Lett Drug Design Discov. (2025) 22:100141. doi: 10.1016/j.lddd.2025.100141 42574925

[25] Spagnolo P Lee JS Sverzellati N Rossi G Cottin V . The lung in rheumatoid arthritis: focus on interstitial lung disease. Arthritis Rheumatol. (2018) 70:1544–54. doi: 10.1002/art.40574 29806092

[26] Nieto MA Rodriguez-Nieto MJ Sanchez-Pernaute O Romero-Bueno F Leon L . Mortality rate in rheumatoid arthritis-related interstitial lung disease: the role of radiographic patterns. BMC Pulmonary Med. (2021) 21:205. doi: 10.1186/s12890-021-01569-5 34193085 PMC8243533

[27] Juge PA Granger B Debray MP Ebstein E Louis-Sidney F Kedra J . A risk score to detect subclinical rheumatoid arthritis-associated interstitial lung disease. Arthritis Rheumatol. (2022) 74:1755–65. doi: 10.1002/art.42162 PMC9828082 35583934

[28] Chang SH Paudel ML McDermott GC Zhang Q Fukui S Kim M . Development of a prediction model for progression of rheumatoid arthritis-associated interstitial lung disease using serologic and clinical factors: the prospective KORAIL cohort. Semin Arthritis Rheumatism. (2025) 73:152729. doi: 10.1016/j.semarthrit.2025.152729 40294559

[29] Severo CR Chomiski C Valle MBD Escuissato DL Paiva EDS Storrer KM . Assessment of risk factors in patients with rheumatoid arthritis-associated interstitial lung disease. Jornal Brasileiro Pneumologia. (2022) 48:e20220145. doi: 10.36416/1806-3756/e20220145 PMC9720882 36477171

[30] Ito Y Ichikawa Y Murashima S Sakuma H Nakajima A . Rheumatoid arthritis disease activity significantly impacts on the severity of interstitial lung disease. Arthritis Res Ther. (2024) 26:95. doi: 10.1186/s13075-024-03333-6 38704556 PMC11069302

[31] Xie S Li S Chen B Zhu Q Xu L Li F . Serum anti-citrullinated protein antibodies and rheumatoid factor increase the risk of rheumatoid arthritis-related interstitial lung disease: a meta-analysis. Clin Rheumatol. (2021) 40:4533–43. doi: 10.1007/s10067-021-05808-2 34189672

[32] Zheng M Lou A Zhang H Zhu S Yang M Lai W . Serum KL-6, CA19-9, CA125 and CEA are diagnostic biomarkers for rheumatoid arthritis-associated interstitial lung disease in the Chinese population. Rheumatol Ther. (2021) 8:517–27. doi: 10.1007/s40744-021-00288-x 33586127 PMC7991038

[33] Guo L Wang J Li J Yao J Zhao H . Biomarkers of rheumatoid arthritis-associated interstitial lung disease: a systematic review and meta-analysis. Front Immunol. (2024) 15:1455346. doi: 10.3389/fimmu.2024.1455346 39534599 PMC11554464

[34] Szekanecz E Sándor Z Antal-Szalmás P Soós L Lakos G Besenyei T . Increased production of the soluble tumor-associated antigens CA19-9, CA125, and CA15-3 in rheumatoid arthritis: potential adhesion molecules in synovial inflammation? Ann N Y Acad Sci. (2007) 1108:359–71. doi: 10.1196/annals.1422.037 17893999

[35] Ba C Jiang C Wang H Shi X Jin J Fang Q . Prognostic value of serum oncomarkers for patients hospitalized with acute exacerbation of interstitial lung disease. Ther Adv Respir Dis. (2024) 18:17534666241250332. doi: 10.1177/17534666241250332 38757948 PMC11102678

[36] Yao C Lu Y Dou D Xia C Ma X Yang Y . Rheumatoid arthritis-associated interstitial lung disease: clinical predictive model and external validation. Respir Res. (2025) 26:331. doi: 10.1186/s12931-025-03416-1 41299487 PMC12659138

