# Supplementary material for: Development and external validation of a clinical-genetic risk identification model for rheumatoid arthritis-associated interstitial lung disease
Source: Front Immunol. 2026 Sep 3;17:1885469. doi: 10.3389/fimmu.2026.1885469 (PMC13582460; doi:10.3389/fimmu.2026.1885469)
Supplement: Supplementary file 1 [file DataSheet1.docx]

Figure S1. Bootstrap validation of the clinical risk identification model.

A: Distribution of optimism (Bootstrap AUC − Test AUC) from 1,000 bootstrap resamples. The mean optimism was 0.0119, indicating minimal overfitting of the model. B: Comparison of AUC values across Bootstrap (internal validation), Test (original sample), and Apparent estimates.

Figure S2 LASSO 10-fold cross-validation for variable selection
